# Preferences and willingness to pay for early childhood healthy lifestyle initiative outcomes: A discrete choice experiment

**DOI:** 10.1111/ijpo.70033

**Published:** 2025-06-12

**Authors:** Vicki Brown, Brittany J. Johnson, Thomas Lung, Alison Hayes, Karen Matvienko‐Sikar, Konsita Kuswara, Elisabeth Huynh

**Affiliations:** ^1^ Deakin Health Economics, Institute for Health Transformation Deakin University Geelong Victoria Australia; ^2^ NHMRC Centre of Research Excellence in Translating Early Prevention of Obesity in Childhood (CRE EPOCH‐Translate) University of Sydney Sydney New South Wales Australia; ^3^ Caring Futures Institute College of Nursing and Health Sciences, Flinders University Tarntanya South Australia Australia; ^4^ Faculty of Medicine and Health, School of Public Health University of Sydney Sydney New South Wales Australia; ^5^ The George Institute for Global Health University of New South Wales Sydney New South Wales Australia; ^6^ School of Public Health University College Cork Cork Ireland; ^7^ Institute for Physical Activity and Nutrition, School of Exercise and Nutrition Sciences Deakin University Geelong Victoria Australia; ^8^ Department of Health Economics Wellbeing and Society College of Health and Medicine, Australian National University Canberra Australian Capital Territory Australia

**Keywords:** early childhood, obesity, preferences, value

## Abstract

**Background:**

Understanding stakeholder preferences and values for early childhood initiatives to support healthy diets, physical activity and reduce sedentary behaviour is key for effective intervention design and resource allocation. This study aims to estimate the preferences for and value of outcomes from the perspectives of parents/caregivers of Australian children aged from birth to 5 years.

**Methods:**

Discrete choice experiment, 466 parent/caregivers recruited from online platform. Participants selected between two healthy lifestyle initiatives or a “neither” option. Initiatives were described by attributes including cost, participation and outcomes. Mixed multinomial logistic models were used to determine preferences and willingness‐to‐pay per annum framed as an increase in income taxes.

**Results:**

Effect on diet was the most important influence on parent/caregiver choice to participate (*p* < 0.01), followed by effect on physical activity (*p* < 0.01), wellbeing (*p* < 0.01) and healthy growth (*p* < 0.01). Parents/caregivers were less sensitive to cost for initiatives aimed at specific children (e.g., targeted initiatives for a priority population). Willingness‐to‐pay estimates ranged from AUD$176 for improved wellbeing to $219 for healthier diets.

**Conclusions:**

Results suggest that leveraging the potential for healthier diets, followed by healthier physical activity behaviours, as a key benefit of participation may be particularly attractive to parents/caregivers. In addition, some level of equity preference could be acceptable to parents/caregivers in the allocation of scarce resources.

## 1 INTRODUCTION

Overweight and obesity in very young children is a global public health issue, experienced by approximately 38 million children aged under 5 years worldwide.^1^ Given the high prevalence, and evidence suggesting that unhealthy weight in childhood tracks into adolescence and adulthood,^2^, ^3^ there is a need for early intervention to avert the health, social and economic costs of overweight and obesity to individuals, communities and society.^4^

There is a growing literature on the effectiveness and cost‐effectiveness of obesity prevention initiatives that encourage and support healthy lifestyles in children before they reach school age.^5^, ^6^, ^7^ These complex initiatives commonly target a broad range of relevant risk factors, such as body weight, physical activity, diet and sedentary behaviour.^8^ As such, there is a diverse range of potential outcomes from these initiatives, with a recent scoping review of outcomes collected and reported in early childhood obesity prevention interventions in children aged from birth to 5 years identifying 221 unique outcomes, across 18 outcome domains.^9^

To fund interventions that are of most value, decision‐makers require information on the preferences and values that beneficiaries and payers place on outcomes from interventions.^10^, ^11^, ^12^ Knowledge of patient and consumer preferences is increasingly recognized as important in the decision‐making process, with growing emphasis on person‐centeredness and empowerment.^13^ Studies have explored, for instance, the preferences for outcomes from integrated care^11^, ^14^ or the value of outcomes in schizophrenia treatment^10^ from the perspective of patients. While incorporating patient preferences for treatment outcomes is a growing field in the medical resource allocation and decision‐making literature,^15^ it has gathered far less attention in the field of public and preventive health.

To date, no study has been conducted to quantify how parents/caregivers value outcomes from early childhood healthy lifestyle initiatives, nor to compare the relative magnitude of the preferences for different outcomes. A Delphi study^16^ conducted as part of the development of a Core Outcome Set for early childhood obesity prevention interventions achieved convergence of opinion from key stakeholders, including parents/caregivers, on the importance of a large number of outcomes. Following the Core Outcome Set methodology rating approach^17^ however, meant the value or relative magnitude of preferences for different outcomes could not be quantified.^16^

The aim of this paper was therefore to estimate the preferences for and value of outcomes from early childhood healthy lifestyle initiatives, from the perspective of parents/caregivers of children aged from birth to 5 years. Information on the value or relative magnitude of preferences from the perspective of parents/caregivers would be useful to decision‐makers, to inform priority‐setting of scarce resources incorporating a person‐centred approach. Defining value from the perspective of parents/caregivers is also important because their engagement in early childhood obesity prevention interventions is critical for success.^18^, ^19^ Evidence suggests that program content and relevance are crucial factors for successful parental engagement in early childhood healthy lifestyle initiatives.^20^, ^21^ Knowledge of the value and relative magnitude of preferences for outcomes could therefore also directly inform both intervention design and implementation, through the incorporation of content and participant engagement techniques accounting for these preferences.

## 2 METHODS

A discrete choice experiment (DCE) was undertaken to understand parent/caregiver preferences and willingness to pay for outcomes from early childhood healthy lifestyle initiatives. A DCE is a quantitative method used in health economics and other fields to elicit preferences from participants on a series of hypothetical choice sets.^22^ DCEs can be used to identify and evaluate the relative importance of aspects of decision making related to health outcomes and health services and are appropriate to evaluate stakeholders' willingness to trade off attributes of multi‐attribute services or products.^23^

The study followed recommended guidelines for the design and reporting of DCEs (Data S1, Supporting Information)^23^, ^24^, ^25^ and was approved by the Deakin University Faculty of Health Human Ethics Advisory Group (HEAG‐H 207_2022).

### 2.1 The choice context

**FIGURE 1:**
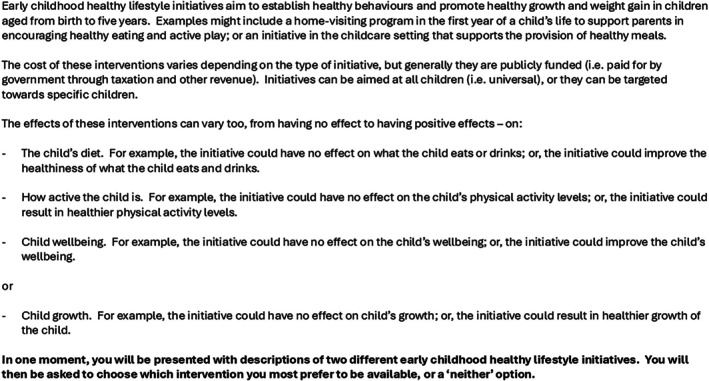
The choice context.

The choice context given to participants is detailed in Figure 1.

Respondents could access a reminder of the choice task, with detailed definitions and examples, while completing the online survey (Data S1).

### 2.2 Sample size and methods for recruitment

While there is no single “gold standard” for sample size estimation for DCEs, a number of approaches have been proposed.^26^, ^27^ These include the rule of thumb proposed by Johnson and Orme.^24^ A sample of approximately 466 parent/caregiver participants is larger than proposed guides (*N* > (460 × (4 × 4))/(12 × 3) = 204),^24^ and sufficient to allow for estimation of robust models and the exploration of heterogeneity in the data. This sample size also accounted for the additional loss of power due to the inclusion of an opt‐out alternative.^28^

Parents/caregivers were recruited using an external panel provider (Pure Profile) in November to December 2023. The panel provider maintains a database of potential survey respondents and emails links to surveys they may be eligible to complete. Potential survey respondents can only participate in each survey once, and the panel provider directly remunerates participants on survey completion (AUD$5.75 per completion). Parents/caregivers were eligible to participate if they were living in Australia, aged 18 years or above, were primary caregiver to a child aged from birth to 5 years, fluent in English and had internet access. Participant quotas were set based on the distribution of children aged from birth to 5 years by Australian state and territory at census date.^29^

### 2.3 Choice of attributes and levels

Participants were presented with a series of choice sets and asked to decide between two unlabelled alternatives (Initiative 1 or Initiative 2) and a “neither” (opt out) option, with options described by a set of attributes. Each participant completed 12 choice sets, corresponding to the median reported in a recent systematic review of DCE studies.^30^ Attributes were assigned levels that vary in each question according to an experimental design, and levels were chosen to encompass the range that is salient to that attribute.^23^ An opt out “neither” option was presented to reflect real life, as early childhood healthy lifestyle initiatives are generally elective programs.^28^

Six attributes were included in the DCE (Table 1 and Data S1). The cost of intervention was included as an attribute, with levels informed by a scoping search of the literature.^7^, ^31^, ^32^, ^33^, ^34^ Cost was framed as a cost to the taxpayer in accordance with the public provision of initiatives and the wording from a recent study.^35^ The population of interest was included as an attribute, given that early childhood healthy lifestyle initiatives can be universal or targeted to specific groups, such as priority populations. Outcomes for inclusion as attributes were informed by the results of a Delphi study, conducted as part of the development of a core outcome set for early intervention trials.^9^, ^16^, ^36^ Briefly, the three round e‐Delphi study was conducted in 2021, and involved 206 researcher, parent/caregiver, health professional, policy‐maker/funder and community/organizational stakeholder participants in Round 1 (with 69% response rate in round 2 and 67% response rate in round 3). Participants were asked to rate the importance of outcomes identified from a published scoping review^9^ on a Likert scale anchored between 1 and 9, where 1–3 signified an outcome that was considered “not that important,” 4–6 “important but not critical” and 7–9 “critically important.” Responses were analysed using mean, median and mean absolute deviation from the median (strength of agreement). Full details of the Delphi study have been published.^16^ Delphi study rankings^16^ and expert judgement were used to refine the potential list of outcomes and levels for the DCE, so as to minimize cognitive burden for participants.^23^ Attribute levels are described in further detail in Data S1.

**TABLE 1:** Attributes, attribute levels and sources.

Attribute	Levels
Cost to the taxpayer (additional cost to you per year, paid as an increase in income taxes)	$6 per year (approx. $0.25 per fortnight)
	$12 per year (approx. $0.50 per fortnight)
	$120 per year (approx. $5 per fortnight)
	$240 per year (approx. $10 per fortnight)
Who can participate in the initiative	All children aged from birth to 5 years (ref)
	Specific children aged from birth to 5 years
Effect on the child's diet	No effect (ref)
	Healthier foods and drinks are consumed
Effect on how active the child is	No effect (ref)
	Healthier amount of time being active
Effect on the child's wellbeing	No effect (ref)
	Improves wellbeing
Effect on the child's growth	No effect (ref)
	Healthier growth pattern

*Note*: Costs are in Australian dollars.

Abbreviation: ref, reference level.

Pre‐testing of the online survey including the DCE was conducted with five participants who were convenience sampled.^37^ Participants either provided verbal or written feedback to the lead author after completing the survey. Minor changes to explanatory text to improve clarity were made prior to piloting of the survey.

### 2.4 Experimental design

The experimental design determined the combinations of attribute levels that formed the alternatives (initiatives) of the choice sets in the DCE. The design followed best practice guidelines^24^ and was generated using Ngene software.^38^ With six attributes, each having either two (five attributes) or four (one attribute) levels, this would result in 128 (2^5^ × 4^1^) potential alternatives and 8128 potential choice sets. A d‐efficient design was selected to generate the smallest possible design sufficient to identify all the necessary parameters.^24^ The design had 24 choice sets, blocked over two versions of the survey and with each respondent answering 12 choice sets. Participants were randomly allocated into a block. We imposed a design constraint that if the attribute for those “who could participate in an initiative” differed between alternatives (e.g., one initiative targeted “specific children” and the other initiative targeted “all children”), then the targeted initiative must cost more than the universal initiative. This constraint was added to avoid dominance of options and to reflect the likely higher cost of initiatives delivered in specific settings or to specific groups.^31^ To generate the initial design, priors were set very close to zero and in the expected direction.^39^ The survey was pilot‐tested with potential participants recruited via the external panel provider (Pure Profile) in order to estimate priors and to further ensure comprehensibility of the tasks (*n* = 45). The d‐efficient design was then updated to reflect priors, and the survey was conducted in the final sample of 466 participants.

### 2.5 Instrument design and administration

Participants were invited to complete the online survey on Qualtrics,^40^ consisting of four sections: (1) eligibility screening and study consent, (2) the DCE, (3) questions about participant views on the importance of early childhood healthy lifestyle initiatives and support for taxation to fund new initiatives (i.e., attitudes), and (4) socio‐demographics. Following recent evidence suggesting that perceived threat (including susceptibility for and severity of condition or risk) may be a determinant of willingness to pay,^12^ participants were asked to rate the extent to which they agreed with three statements on the perceived importance of child obesity on a five‐point scale (where 1 represented “not at all” and 5 represented “very much”) adapted from a previous study.^41^ Participants were also asked how worried they were about their child experiencing overweight or obesity and how worried adults in general should be about child overweight and obesity (where 1 represented “not at all worried” and 5 represented “extremely worried”).^41^ In addition, respondents were asked how supportive they were of governments imposing taxes to fund initiatives, such as healthy lifestyle initiatives (where 1 represented “strongly opposed” and 5 represented “strongly supportive”).

Participant socio‐demographics included age, gender identity, education level, state of residence, postcode, language/s spoken at home, household composition and annual household income. Questions were based on those used in the Australian Census^29^ wherever possible. Socioeconomic position was determined using the Socio‐Economic Indexes for Areas Index of Relative Social Disadvantage (SEIFA IRSD), to categorize by postcode into SEIFA IRSD quintile.^29^

### 2.6 Statistical analyses

The statistical analysis plan was developed following best practice guidelines for the analysis of DCEs.^42^ Analyses were designed and reported according to the ESTIMATE checklist (Data S1).

Data were imported into Stata version 17^43^ for quality checking, cleaning and reordering to obtain stacked choice data (36 cases per participant, comprising 12 choice tasks × 3 alternatives per task). The quality of participant data was checked by assessing the time taken for completion (mean, median time in minutes) and presence of “straight‐lining” responses to the choice tasks.^44^ Participants completing the survey in less than one‐third of the median survey completion time were excluded from analysis, and resampling occurred (*n* = 13).^44^ Straight‐lining was examined by estimating the mean response for all choice tasks and participants considered to be straight‐lining (options 1 or 2) were excluded from analysis and resampled.^44^ Six participants selected the opt‐out option across all choice sets. This behaviour may indicate straight‐lining, protest voting (not wanting to choose an intervention), or simply non‐traders who consistently do not prefer any of the options offered. As such, we retained these responses in the sample for analysis. Sensitivity analysis showed that excluding these individuals did not alter the findings. NLogit version 6 was used for statistical analysis.^45^ Multinomial logit (MNL) models were estimated allowing for hypothesized cost interactions and sociodemographic interactions, based on random utility theory.^27^ MNL analysis results are included in Data S1. Given MNL models assume homogeneous preferences across individuals, mixed multinomial logit models (MMNL) where one or more parameter estimates are randomly distributed among individuals are reported in this paper.^39^ Categorical attributes (who can participate in the initiative, and the effects on diet, activity, wellbeing and growth) were dummy coded with reference levels set to interpret variables as preference utility (Table 1). An interaction effect between cost and who can participate in the initiative was included to identify the additional influence of both attributes on choice. Sociodemographic variables (income, age, gender, language spoken at home, number of adults and children in the household) hypothesized to influence choice were interacted with the alternative specific constant in the model. Categorical sociodemographic variables were dummy coded for the interaction analyses (Data S1). These were added to the model if they improved model fit and were statistically significant (*p* < 0.05).

The final systematic utility function was specified as:

U(Initiative 1) = β_1_Cost + β_2_Popn + β_3_Diet + β_4_Activity + β_5_Wellbeing + β_6_Growth + β_7_Cost‐population.

U(Initiative 2) = β_1_Cost + β_2_Popn + β_3_Diet + β_4_Activity + β_5_ Wellbeing + β_6_Growth + β_7_Cost‐population.

U(Neither) = β_8_ + β_9_Income(low) + β_10_Income(high) + β_11_Female.

The coefficients β_1_‐β_6_ indicate the average relative weight placed on a certain attribute (level).^46^ The constant β_8_ was included as the utility for the “neither” option. Income and gender identity were interacted with the neither option. The coefficients β_9_, β_10_ and β_11_ indicate the weight towards choosing the “neither” option based on low income, high income, and participant gender identity (female), respectively. The final MMNL model included random (popn, diet, activity, wellbeing, growth and the interaction term (normal distributions); cost (constrained triangular distribution)) and non‐random parameters (alternative specific constant, low and high income and female).^47^ The model was run using 1000 Halton draws. Coefficients, 95% confidence intervals and *p* values are presented, with significance set at 0.05. All attributes except cost were dummy coded, with positive coefficients indicating that participants value a particular attribute level over the reference level, and with a larger positive magnitude indicating a stronger preference for that attribute. Conversely, negative coefficients indicate that participants have less preference or are more averse to the attribute level compared to its reference level.

Inclusion of the cost attribute allowed for estimation of willingness to pay, an estimate of the highest price an individual is willing to accept to pay for a good or service.^48^ Marginal willingness to pay was estimated as the ratio of the change in marginal utility of attribute *k* to the change in marginal utility of the cost attribute,^27^ using the Delta method.^39^ Interpretation of this estimate should consider the framing of cost to the participant as “additional cost to you per year, paid as an increase in income taxes” given the publicly funded nature of early childhood healthy lifestyle initiatives (i.e., as a marker of parent acceptability for these initiatives). It should also be noted that willingness to pay is estimated in the preference space, and not in the “willingness to pay space” meaning that estimates should be interpreted in relation to the magnitude of attribute preferences.^49^ Model goodness of fit was assessed using the log likelihood ratio index, McFadden's pseudo‐R‐squared and Akaike's information criterion (AIC).

A secondary analysis explored how attitudes are correlated with choice and added attitudinal variables to the model if they were statistically significant (*p* < 0.05) (Data S1).

## 3 RESULTS

Four‐hundred and sixty‐six participants completed the survey resulting in 5592 choice observations for final analysis. Because participants could only progress to the next choice task on completion of each section of the survey, there was no missing data in completed records. The total proportion of “neither” choices was 8.8% (490 neither choices from 5592 choice observations). The final 466 participants were predominantly female (68%), had completed higher than a Year 12 (high school) level of education (81%) and spoke only English at home (82%) (Table 2).

**TABLE 2:** Descriptive characteristics of survey participants (*n* = 466).

Characteristic	Count (%)
Age	
18–24 years	33 (7%)
25–34 years	181 (39%)
35–44 years	180 (39%)
45–54 years	57 (12%)
55+ years	15 (3%)
Gender	
Male	149 (32%)
Female	317 (68%)
State of residence	
Australian Capital Territory	9 (2%)
New South Wales	144 (31%)
Queensland	92 (20%)
Victoria	116 (25%)
Tasmania	8 (2%)
Northern Territory	6 (1%)
Western Australia	56 (12%)
South Australia	35 (8%)
Education	
Year 11 or below	32 (7%)
High school certificate	52 (11%)
Certificate/Diploma/Undergraduate degree	322 (69%)
Postgraduate degree (Masters/PhD)	59 (12%)
Prefer not to say	1 (<1%)
Language other than English spoken at home	
No, only English spoken at home	384 (82%)
Yes, another language spoken at home	82 (18%)
No adults normally reside in household	
1	55 (12%)
2	364 (78%)
3	29 (6%)
4+	18 (4%)
No children aged 6–17 years normally reside in household	
0	237 (51%)
1	139 (30%)
2	63 (14%)
3	22 (5%)
4+	5 (1%)
No children aged 0–5 years normally reside in household	
1	314 (67%)
2	130 (28%)
3	20 (4%)
4+	2 (0.4%)
Annual household income before tax (AUD)	
$0 or nil	2 (0.6%)
$1 to $51 999 ($1–$999 per week)	47 (10%)
$52 000 to $155 999 ($1000–$2999 per week)	247 (53%)
$156 000 to $259 999 ($3000–$4999 per week)	126 (27%)
$260 000 to $415 999 ($5000–$7999 per week)	16 (3%)
$416 000 or more ($8000 or more per week)	1 (0.2%)
Rather not say	27 (5%)
Socioeconomic Index For Areas‐Index of Relative Disadvantage	
Quintile 1 (most disadvantaged)	62 (13%)
Quintile 2	88 (19%)
Quintile 3	89 (19%)
Quintile 4	101 (22%)
Quintile 5 (least disadvantaged)	126 (27%)
Ever participated in programs or services to support healthy behaviours and/or growth	
Yes	100 (21%)
No	358 (77%)
Rather not say	8 (2%)

Most parent/caregivers agreed or strongly agreed that childhood obesity is serious (*n* = 368, 79%), can cause harm in childhood (*n* = 397, 85%) and can lead to life‐threatening diseases in later life (*n* = 394, 84%) (Data S1). Most parent/caregivers were not worried at all or only a little bit worried about their own child experiencing overweight or obesity (*n* = 281, 61%; Data S1). The majority of parent/caregivers were, however, moderately to extremely worried about children in general experiencing overweight or obesity (*n* = 384, 82%). Seventy‐three parent/caregiver participants (16%) were opposed or strongly opposed to the government imposing taxes to fund new initiatives such as early childhood healthy lifestyle initiatives (compared with 36% somewhat supportive, 32% supportive, 17% strongly supportive).

**FIGURE 2:**
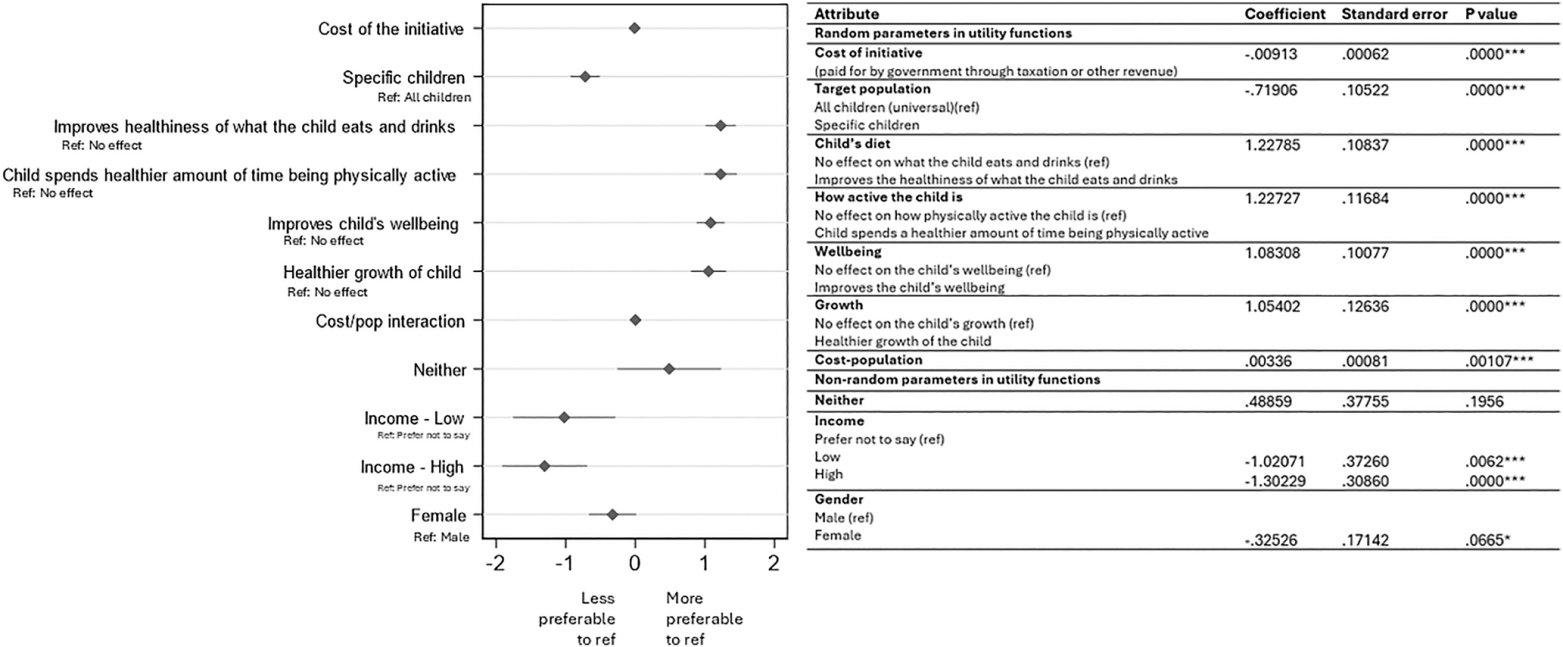
Forest plot and mixed multinomial logit model analysis results. ***Significant at 1% level; **significant at 5% level; *significant at 10% level.

All attributes were found to significantly influence parent/caregivers choice to participate in an early childhood healthy lifestyle initiative (Figure 2). Effect on diet was the most important (*β* = 1.228, *p* = <0.001) with parents/caregivers favouring initiatives that resulted in healthier foods and drinks being consumed. This was followed by effect on physical activity (*β* = 1.227, *p* = <0.001), wellbeing (*β* = 1.083, *p* = <0.001), and healthy growth (*β* = 1.054, *p* = <0.001). A negative coefficient for target population suggests that initiatives to specific children (targeted initiatives) were less preferred compared to initiatives aimed at the general population (universal initiatives). On average, parents/caregivers prefer less costly initiatives. The significant and positive interaction effect between cost and target population (cost‐population) suggests that parents/caregivers were less sensitive to cost for initiatives aimed at specific children (targeted initiatives). Household income had a significant influence on whether parents/caregivers chose an initiative versus the neither option. Gender had a significant influence on whether parents/caregivers chose an initiative versus the neither option at the 10% level (*p* = 0.06). In particular, females (compared to males) and those with higher household incomes were less likely to choose the neither option (i.e., are more likely to choose a healthy lifestyle initiative) (Figure 2).

Coefficients in Figure 2 are used to calculate the willingness to pay estimates presented in Table 3. Willingness to pay for outcomes from initiatives ranged from $175.83 (unit change in wellbeing) to $219.15 (unit change in healthiness of what the child eats and drinks).

**TABLE 3:** Willingness to pay estimates for each unit increase in outcome attribute.

Outcome attributes	Willingness to pay ($AUD)	Standard deviation
Effect on the child's diet	$219.15*	198.45
Effect on how active the child is	$201.23*	153.09
Effect on the child's wellbeing	$175.83*	138.47
Effect on the child's growth	$185.15*	204.86

Abbreviations: AUD, Australian dollars; 95% CI, 95% confidence interval.

*Significant at 1% level.

Results from the secondary analysis (reported in Data S1) suggest that parents/caregivers who are more worried about children experiencing overweight or obesity are less likely to prefer the neither option (i.e., more likely to choose a healthy lifestyle initiative). In addition, parents/caregivers who were more supportive of taxes to fund initiatives are also less likely to prefer the neither option (i.e., more likely to choose a healthy lifestyle initiative). There were no material differences in utility weights for attributes as compared to primary analysis results.

## 4 DISCUSSION

This study aimed to estimate parents/caregivers preferences for and value of outcomes from early childhood healthy lifestyle initiatives. Of the outcomes presented within our study, parents/caregivers preferred initiatives resulting in healthier diets, followed by initiatives resulting in healthier physical activity behaviours, wellbeing, and growth. This provides useful information for those designing and implementing early childhood lifestyle initiatives, many of which aim to influence a range of risk‐factor related outcomes.^9^ Research shows that considering and incorporating factors that are important to parents/caregivers in designing initiatives can improve their reach and impact by boosting engagement, interaction and retention.^20^, ^50^ Using the findings from this study on parent/caregiver preferences for outcomes, interventionists could tailor the design, content and/or promotion of initiatives in order to maximize parental interest and participation. While most early childhood healthy lifestyle initiatives necessarily focus on more than one risk factor given the aetiology of obesity is multifactorial, our results suggest that leveraging the potential for healthier diets, followed by healthier physical activity behaviours, as a key benefit of participation may be particularly attractive to Australian parents/caregivers of children aged from birth to 5 years.

An efficient allocation of resources requires rigorous information on how potential beneficiaries and payers value health initiatives.^12^ Parents/caregivers in our study in general preferred less costly, universal child healthy lifestyle initiatives. While this in itself is not a surprising finding, the significant interaction effect observed between cost and the target population suggests that some level of equity preference or weighting might be considered appropriate or acceptable to parents/caregivers in the allocation of scarce resources to early childhood healthy lifestyle initiatives. To date, there have been limited published economic evaluations of healthy lifestyle initiatives conducted in very young children incorporating equity considerations into their analyses.^51^ More generally, the inclusion of equity value judgements into health economic evaluation is a growing field, and this is an important area for future research.^52^ A better understanding of parent/caregiver understanding and perceptions of proportionate universalism, or universal action with a scale and intensity proportionate to the level of disadvantage,^53^ is also required. This could be further explored using techniques such as “think aloud,” where participants verbalize their thinking while making choices in order to explore whether preferences are consistent with utility theory.^54^

In our study, male identifying parents/caregivers were less likely to prefer an initiative versus the neither option, with this finding further confirming the well‐documented challenges in regard to the inclusion of fathers and male caregivers in childhood obesity prevention interventions.^55^ Participants in our study with higher household incomes were also more likely to select an initiative versus the neither option. Challenges with reach and inclusion of different demographic groups, including low socioeconomic and other priority populations, in some early childhood healthy lifestyle initiatives have been documented.^56^ Future research, specifically targeting priority populations for recruitment to studies examining choice preferences for child healthy lifestyle initiatives, is recommended to better understand the preferences and values of these important subgroups.

Comparison with the literature is challenging, given that most previous DCE studies have defined attributes in terms of the characteristics of initiatives, rather than in terms of outcomes.^14^ We are not aware of any studies examining preferences for outcomes from obesity prevention initiatives, or from initiatives in very young children. A recent multi‐country study examined relative preferences for outcomes of integrated care in those with multimorbidity, identifying the wellbeing outcome as the most important outcome among participants.^14^ Our study differed in that parent/caregiver preferences were elicited rather than eliciting preferences from children themselves, and child wellbeing was not the most preferred outcome in our study. Similar to the finding from Molken et al.^14^ however, the positive and significant coefficient for wellbeing in our study highlights the importance of measuring the value of early childhood healthy lifestyle initiatives beyond physical health to reflect the broader benefit of these initiatives. While child wellbeing is generally not the primary outcome of early childhood healthy lifestyle initiatives,^9^ it was identified as a core outcome recommended for collection in a recently developed core outcome set.^16^ In order to further reflect the potential broader benefits of early childhood healthy lifestyle initiatives, more evidence of the impact of early intervention on outcomes such as education and socialization, material security and social inclusion is also needed.^57^

Previous studies have explored the willingness to pay for childhood obesity prevention in parents^58^ and the general public^59^; or commissioners' willingness to pay for community‐based childhood obesity prevention programmes that specifically resulted in additional portions of fruits and vegetables eaten per day.^60^ Direct comparison of our findings with this literature is challenging, given differing aims and methodologies used. For example, the German study by Kesztyus et al.^58^ estimated a mean willingness to pay of EUR23.04 per month (equivalent of EUR276.48 per year; approx. AUD444.00 [EUR1.00 = AUD1.605]) to reduce the incidence of childhood overweight/obesity by half, by asking parents to pick the monetary category they were willing to pay (ranging from “EUR1‐EUR5” to “EUR301‐EUR500” per month). The study by Webb et al.^60^ undertook a DCE with commissioners and decision makers in the UK, estimating their willingness to pay in annual running costs for childhood obesity programs. Results suggested that participants were willing to pay an additional GBP16,600 per year for a program that increased the daily fruit and vegetable intake for each child involved by one portion (approx. AUD31,669; GBP1.00 = AUD1.908). The authors noted that commissioners and decision makers were willing to pay large amounts of money for childhood obesity programs in the UK, but that it was out of scope to assess whether this willingness to commit substantial funding amounts was likely to result in an optimal use of resources.^60^

While our willingness to pay estimates are not directly comparable, they do indicate a substantial willingness to pay from parents/caregivers through the Australian taxation system for healthy lifestyle initiatives that improve healthy diets or activity behaviours in particular. It should however be noted that these willingness to pay values may not be reflective of the broader Australian populations values in a publicly funded healthcare system, given that potential beneficiaries of an initiative may value it differently as compared to non‐users. It should also be noted that stated preference studies may overestimate willingness to pay due to factors such as hypothetical bias.^61^ More research could be undertaken to explore the willingness to pay for early childhood healthy lifestyle initiatives as compared to other publicly funded initiatives, from the perspective of the whole population and incorporating strategies to reduce hypothetical bias.^62^

Strengths of our study include attribute and level selection incorporating rigorous evidence from the literature, extensive pilot testing for comprehension and to estimate priors for analysis and a sufficient sample size that was above the median for DCEs.^30^ Study limitations include concerns regarding cognitive burden limiting the set of attributes and levels that could be included in our DCE.^63^ We attempted to circumvent this limitation, however, through the selection of salient outcome attributes in our DCE using the results from a published Delphi study.^16^ Further limitations include low participant representation from the lowest quintile of socioeconomic deprivation (*n* = 62, 13%) and high representation from households where only English is spoken at home (*n* = 384, 82%). In addition, our results indicate the preferences of parents/caregivers but do not incorporate the preferences of other stakeholders, for instance, funders or decision makers. Future work will explore the preferences for outcomes from other perspectives important to the success of early childhood healthy lifestyle initiatives.

## 5 CONCLUSIONS

Understanding parent/caregiver preferences for outcomes is critical for the optimal design of acceptable interventions to improve the health of children. Findings from our DCE indicate that parents/caregivers prefer initiatives resulting in healthier diets, followed by initiatives resulting in healthier physical activity behaviours, wellbeing and growth. Our findings also suggest that parents/caregivers may find it acceptable to incorporate some degree of equity preference or weighting when allocating limited resources for early childhood healthy lifestyle initiatives. This information is useful in the design and implementation of initiatives and should be considered in the allocation of scarce resources to improving the health of young children. More research is, however, required to investigate the preferences of priority populations and the preferences of the broader population for taxpayer‐funded initiatives.

## AUTHOR CONTRIBUTIONS

VB conceived the study, with expert input from all authors. VB designed the discrete choice experiment, and all authors reviewed the selection of attributes and levels. VB drafted the survey, with review by all authors. VB cleaned and conducted the data analysis, with supervision and expert guidance from EH. VB drafted the paper, and all authors provided extensive review and editing.

## CONFLICT OF INTEREST STATEMENT

The authors declare no conflicts of interest.

## Supporting information


**Data S1.** Supporting Information.

## Data Availability

The datasets generated and analysed during the current study are available from the corresponding author on reasonable request.
