# Supplementary material for: Preferences and willingness to pay for early childhood healthy lifestyle initiative outcomes: A discrete choice experiment
Source: Pediatr Obes. 2025 Jun 12;20(9):e70033. doi: 10.1111/ijpo.70033 (PMC12329628; doi:10.1111/ijpo.70033)
Supplement: Supplementary file 1 — Data S1. Supporting Information. [file IJPO-20-e70033-s001.pdf]

Supplementary materials

Preferences for early childhood healthy lifestyle initiative outcomes –a discrete choice experiment

Vicki Brown[1,2], Brittany J Johnson[2,3], Thomas Lung[2,4,5], Alison Hayes[2,4], Karen Matvienko-Sikar[2,6], Konsita Kuswara[2,7], Elisabeth Hyunh[8]

1 Deakin University, Geelong, Australia, Deakin Health Economics, Institute for Health Transformation

2 NHMRC Centre of Research Excellence in Translating Early Prevention of Obesity in Childhood (CRE EPOCH-Translate), University of Sydney, Sydney, Australia

3 Flinders University, College of Nursing and Health Sciences, Caring Futures Institute, Tarntanya, Australia

4 University of Sydney School of Public Health, Faculty of Medicine and Health

5 The George Institute for Global Health, UNSW Sydney

6 School of Public Health, University College Cork, Cork, Ireland

7 Deakin University, Geelong, Australia, School of Exercise and Nutrition Sciences, Institute for Physical Activity and Nutrition

8 Department of Health Economics Wellbeing and Society, College of Health and Medicine, Australian National University.

Corresponding author:

Dr Vicki Brown

Deakin Health Economics, Institute for Health Transformation, Deakin University, 221 Burwood Highway, VIC, Australia

+61 3 9244 6432

victoria.brown@deakin.edu.au

Competing interests: The authors declare no competing interests

Supplementary File 1 - Checklist for conjoint analysis applications in health care [1]

Checklist item	Page no.
1. Was a well-defined research question stated and is conjoint analysis an appropriate method for answering it?<br>1.1 Were a well-defined research question and a testable hypothesis articulated?<br>1.2 Was the study perspective described, and was the study placed in a particular decision-making or policy context?<br>1.3 What is the rationale for using conjoint analysis to answer the research question?	3-4
2. Was the choice of attributes and levels supported by evidence?<br>2.1 Was attribute identification supported by evidence (literature reviews, focus groups, or other scientific methods)?<br>2.2 Was attribute selection justified and consistent with theory?<br>2.3 Was level selection for each attribute justified by the evidence and consistent with the study perspective and hypothesis?	5-6<br>Supp File 3
3. Was the construction of tasks appropriate?<br>3.1 Was the number of attributes in each conjoint task justified (that is, full or partial profile)?<br>3.2 Was the number of profiles in each conjoint task justified?<br>3.3 Was (should) an opt-out or a status-quo alternative (be) included?	5-7
4. Was the choice of experimental design justified and evaluated?<br>4.1 Was the choice of experimental design justified? Were alternative experimental designs considered?<br>4.2 Were the properties of the experimental design evaluated?<br>4.3 Was the number of conjoint tasks included in the data-collection instrument appropriate?	5-7
5. Were preferences elicited appropriately, given the research question?<br>5.1 Was there sufficient motivation and explanation of conjoint tasks?<br>5.2 Was an appropriate elicitation format (that is, rating, ranking, or choice) used? Did (should) the elicitation format allow for indifference?<br>5.3 In addition to preference elicitation, did the conjoint tasks include other qualifying questions (for example, strength of preference, confidence in response, and other methods)?	5-7
6. Was the data collection instrument designed appropriately?<br>6.1 Was appropriate respondent information collected (such as sociodemographic, attitudinal, health history or status, and treatment experience)?<br>6.2 Were the attributes and levels defined, and was any contextual information provided?<br>6.3 Was the level of burden of the data-collection instrument appropriate? Were respondents encouraged and motivated?	5-7
7. Was the data-collection plan appropriate?	5-7

7.1 Was the sampling strategy justified (for example, sample size, stratification, and recruitment)?<br>7.2 Was the mode of administration justified and appropriate (for example, face-to-face, pen-and-paper, web-based)?<br>7.3 Were ethical considerations addressed (for example, recruitment, information and/or consent, compensation)?	
8. Were statistical analyses and model estimations appropriate?<br>8.1 Were respondent characteristics examined and tested?<br>8.2 Was the quality of the responses examined (for example, rationality, validity, reliability)?<br>8.3 Was model estimation conducted appropriately? Were issues of clustering and subgroups handled appropriately?	8-10
9. Were the results and conclusions valid?<br>9.1 Did study results reflect testable hypotheses and account for statistical uncertainty?<br>9.2 Were study conclusions supported by the evidence and compared with existing findings in the literature?<br>9.3 Were study limitations and generalizability adequately discussed?	10-12<br>12-15
10. Was the study presentation clear, concise, and complete?<br>10.1 Was study importance and research context adequately motivated?<br>10.2 Were the study data-collection instrument and methods described?<br>10.3 Were the study implications clearly stated and understandable to a wide audience?	3-4<br>4-10<br>10-12<br>12-16

Checklist for reporting discrete choice experiments in health [2]

**Item**		**Page and paragraph**
Purpose and rationale		
1	Describe the real-world context and decision-maker that the hypothetical choice context seeks to replicate or inform	3-4
2	Provide a rationale for using a DCE to answer the research question	3-4
Attributes and levels		
3	Describe how attributes and levels were derived (e.g. literature review, interviews, focus groups, expert input)	6-7
4	Provide the final list of attributes and levels	Table 1, 6-7
Experimental design		
5	Report the number of alternatives per choice set and whether they were labelled or unlabelled	7-8
6	Describe response options (e.g. forced choice, opt-out, status quo)	7-8
7	Describe the type of experimental design (e.g. orthogonal, D-efficient, Bayesian efficient, partial profile)	7-8
8	Describe which effects are identified in the design (e.g. main effects, higher order interactions, functional form)	9-11
9	Describe the number of choice sets, blocks and choice sets per block	7-8
10	Indicate how the experimental design was obtained (software, catalogue, other)	8-9
Survey design		
11	Provide a sample choice set and the instructions and background information given to respondents (e.g. providing the survey as an appendix)	Supplementary file 2
12	Report any randomisation (e.g. choice set order, attribute order, alternative order, framing effects)	7-8
13	Describe what was checked in piloting (e.g. understanding, respondent burden, timing, wording)	8
14	Report whether information from the pilot was used to update the experimental design (e.g. priors, functional form of attributes) or survey design	8
Sample and data collection		
15	Report respondent inclusion/exclusion criteria	5-6
16	Describe how data were collected (e.g. mail, personal interview, web survey)	8
17	Report the response rate or cooperation rate, if possible	11
18	Report the final sample size and how the sample size was determined	11
19	Describe respondent characteristics and representativeness of target population, if known	Table 2, 11-12
Econometric analysis		

20	Indicate coding of data (e.g. effects, dummy, continuous) including definitions	9-11
21	Report whether any respondents were removed and why (e.g. suspected fraudulent responses, rationality tests)	9-11
22	Provide the rationale for model choice and assumptions	9-11
23	Report model specification	9-11
Reporting of results		
24	Report the model performance, goodness of fit (if comparing models)	11
25	Describe methods used for analysis of model results (e.g. calculation of marginal rate of substitution, attribute relative importance, welfare gain)	11
26	Report measures of precision for the output(s) of interest (e.g. confidence intervals) and how these were derived	11

Supplementary File 2 - The survey instrument

Page 1

What impacts of early childhood healthy lifestyle initiatives are important to parents?

Early childhood (birth to 5 years) is a critical time to encourage and support children to live healthy and happy lives. Early childhood healthy lifestyle initiatives aim to establish healthy behaviours and promote healthy growth and weight gain in children aged from birth to five years.

Our study is designed to explore the different impacts that these initiatives may have – including on a child's diet, physical activity, wellbeing and healthy growth.

In this survey, you will be asked about which features of early childhood healthy lifestyle initiatives are most important to you. Features of two early childhood healthy lifestyle initiatives will be described. You will be asked to choose the one you most prefer to be available as an initiative - as a parent, but also more broadly when thinking about what should be available as an initiative for other children.

You will be asked to complete this "choice task" 12 times. The survey should take no longer than 15-20 minutes in total to complete.

Your answers will help us to better understand the importance of these initiatives and support future policy and practice decisions.

Thank you for your participation. This study is being undertaken by researchers at Deakin University, University of Sydney, Australian National University, University College Cork and Flinders University. The Deakin Human Research Ethics Committee (HREC) has approved this study (207_2022). If you have any questions or would like further information please contact Vicki Brown: victoria.brown@deakin.edu.au.

Page 2

Important information on your participation in our study is available in the downloadable Plain Language Statement. This information explains the study procedure and how your data will be used and stored. Please take the time to download and read this information.

Plain Language Statement (hyperlink)

Have you read and understood the Plain Language Statement and do you agree to take part in this project according to the conditions described?

- ◯ Yes, I agree to take part according to the conditions in the Plain Language Statement and acknowledge that the researcher has agreed not to reveal my identity and personal details, including where information about this project is published, or presented in any public form.
- ◯ No, I do not agree to participate.

Page 3

Before you are directed to the study survey, please answer the following questions to ensure you are eligible to participate.

Are you a parent or primary caregiver of at least one child aged five years or younger, residing in Australia and comfortable reading English?

- ◯ Yes
- ◯ No

Page 4

What state/territory do you currently reside in?

- ◯ ACT
- ◯ NSW
- ◯ QLD
- ◯ VIC
- ◯ TAS
- ◯ NT
- ◯ WA
- ◯ SA

Page 5

The survey has three sections.

Section A consists of the "choice task". Features of two different early childhood healthy lifestyle initiatives will be described. You will be asked to choose which option you most prefer. Please choose the one you most prefer to be available as an initiative for children aged from birth to five years. If you do not prefer either, you can select a 'neither' option. You will complete this task 12 times.

Section B will ask some questions about your views on the importance of early childhood healthy lifestyle initiatives and obesity prevention.

Section C will ask some questions about you and your household.

Thank you for participating in this study.

Page 6

Early childhood healthy lifestyle initiatives aim to establish healthy behaviours and promote healthy growth and weight gain in children aged from birth to five years.

Examples might include a home-visiting program in the first year of a child's life to support parents in encouraging healthy eating and active play; or an initiative in the childcare setting that supports the provision of healthy meals.

The cost of these initiatives varies depending on the type of initiative, but generally, they are publicly-funded (i.e. paid for by government through taxation and other revenue).

Initiatives can be aimed at all children (i.e. universal), or they can be targeted towards specific children.

The impacts of these initiatives can vary too, from having no effect to having positive effects, on:

- The child's diet. For example, the initiative could have no effect on what the child eats or drinks; or, the initiative could improve the healthiness of what the child eats and drinks.

- How active the child is. For example, the initiative could have no effect on how physically active the child is; or, the initiative could result in the child spending a healthier amount of time being physically active.

- Child wellbeing. For example, the initiative could have no effect on the child's wellbeing; or, the initiative could improve the child's wellbeing.

or

- Child growth. For example, the initiative could have no effect on the child's growth; or, the initiative could result in healthier growth of the child.

In one moment, you will be presented with descriptions of two different early childhood healthy lifestyle initiatives. You will then be asked to choose which intervention you most prefer to be *available*, or a 'neither' option.

Page 7

Here is an example:

The table describes two early childhood healthy lifestyle initiatives. Which of these early childhood healthy lifestyle initiatives do you most prefer?

	Initiative 1	Initiative 2	Neither
Cost to the taxpayer (additional cost to you per year, paid as an increase in income taxes)	$240 per year (approx. $10 per fortnight)	$120 per year (approx. $5 per fortnight)	
Who can participate in the initiative	Specific children aged from birth to five years	Any child aged from birth to five years	
Effect on the child's diet	No effect	Healthier foods and drinks are consumed	Neither
Effect on how active the child is	Healthier amount of time being active	No effect	
Effect on the child's wellbeing	No effect	Improves wellbeing	
Effect on the child's growth	No effect	Healthier growth pattern	
	○	○	○

After you have read the descriptions, select whether you prefer Initiative 1, Initiative 2 or Neither

In this example:

Initiative 1:
- costs the taxpayer $240 per year

- is targeted towards specific children

- and, increases the child's time spent being physically active

Initiative 2:
- costs the taxpayer $120 per year
- any child can participate (i.e. universal)
- improves the healthiness of what the child eats and drinks
- increases the child's wellbeing
- and, results in healthier growth of the child.

After reading the descriptions for Initiative 1 and Initiative 2, all you need to do is select which you prefer - either Initiative 1, Initiative 2 or Neither.

If you would like, please click on the link below and a reminder of the choice task will open in another window. You can refer to this as you go, if you are unsure of any of the definitions. Once this window opens, don't forget to come back here to finish the survey!

Let's get started.

(If participant clicks on link, reminder of the choice task is displayed)

This is a reminder of the "choice task". Please keep this window open, and refer to the *definitions* and *examples* should you need.

Early childhood healthy lifestyle initiatives aim to establish healthy behaviours and promote healthy growth and weight gain in children aged from birth to five years.

Examples might include a home-visiting program in the first year of a child's life to support parents in encouraging healthy eating and active play; or an initiative in the childcare setting that supports the provision of healthy meals.

The cost of these initiatives varies depending on the type of initiative, but generally, they are publicly-funded (i.e. paid for by government through taxation and other revenue).

Initiatives can be aimed at all children (i.e. universal), or they can be targeted towards specific children.

The impacts of these initiatives can vary too, from having no effect to having positive effects, on:

- The child's diet. For example, the initiative could have no effect on what the child eats or drinks; or, the initiative could improve the healthiness of what the child eats and drinks.

- How active the child is. For example, the initiative could have no effect on how physically active the child is; or, the initiative could result in the child spending a healthier amount of time being physically active.

- Child wellbeing. For example, the initiative could have no effect on the child's wellbeing; or, the initiative could improve the child's wellbeing.

or

- Child growth. For example, the initiative could have no effect on the child's growth; or, the initiative could result in healthier growth of the child.

In one moment, you will be presented with descriptions of two different early childhood healthy lifestyle initiatives. You will then be asked to choose which intervention you most prefer to be available, or a 'neither' option.

Definitions provided as hover text for fields in blue throughout the entire survey include:

Active- This includes structured physical activity like swimming lessons; as well as infant time and active play.

Available – available means offered. Regardless of whether individual parents choose to participate with their children or not.

Growth – Increases in height and weight as a child grows.

Healthier growth – A child that is growing well. For example, a child that is not growing too slowly (i.e. underweight) or too fast (i.e. overweight or obesity).

Healthiness – For example, the child eats more nutritious foods and drinks.

Parent – A mother, father, guardian or other person who fulfills the parental responsibilities of caring for a child.

Specific children – For example, children living in low socioeconomic areas or children from families that speak languages other than English at home.

Wellbeing – Wellbeing is a broad concept, and is generally understood to encompass health and safety, material security, education and socialisation, sense of being loved and valued, sense of being included in families and societies, quality of life and mental health.

Choice tasks 1-12, programmed using Qualtrics conjoint analysis feature [3] and design generated in Ngene software [4]

The table describes two early childhood healthy lifestyle initiatives. Which of these early childhood healthy lifestyle initiatives do you most prefer?

	Initiative 1	Initiative 2	Neither
Cost to the taxpayer (additional cost to you per year, paid as an increase in income taxes)	$6 per year (approx. $0.25 per fortnight)	$120 per year (approx. $5 per fortnight)	Neither
Who can participate in the initiative	Any child aged from birth to five years	Specific children aged from birth to five years	
Effect on the child's diet	No effect	Healthier foods and drinks are consumed	
Effect on how active the child is	Healthier amount of time being active	No effect	
Effect on the child's wellbeing	No effect	Improves wellbeing	
Effect on the child's growth	Healthier growth pattern	No effect	
	○	○	○

>>

Page 20

We would now like to hear your views on the importance of early childhood healthy lifestyle initiatives and obesity prevention.

Please rate the extent to which you agree with the following statements.

Childhood obesity is serious

- ○ I do not agree at all
- ○ I am not sure
- ○ I somewhat agree
- ○ I agree
- ○ I strongly agree

Childhood obesity can cause harm in childhood

- I do not agree at all
- I am not sure
- I somewhat agree
- I agree
- I strongly agree

Childhood obesity can lead to life-threatening diseases later in life

- I do not agree at all
- I am not sure
- I somewhat agree
- I agree
- I strongly agree

How worried are you about your child (aged from birth to five years) experiencing overweight or obesity?

- Not worried at all
- A little bit worried
- Moderately worried
- Very worried
- Extremely worried

How worried should parents be about their children experiencing overweight or obesity?

- ◯ Not worried at all
- ◯ A little bit worried
- ◯ Moderately worried
- ◯ Very worried
- ◯ Extremely worried

How supportive are you of the government imposing taxes to fund new initiatives such as early childhood healthy lifestyle initiatives?

- ◯ Strongly opposed
- ◯ Opposed
- ◯ Somewhat supportive
- ◯ Supportive
- ◯ Strongly supportive

Page 21

Please answer the following demographic questions. You will not be asked to provide any information which could be used to identify you.

What is your age? (dropdown)

What is your gender identity? (dropdown)

What is your postcode? (dropdown)

What is your highest level of educational attainment? (dropdown)

Do you use a language other than English at home? If more than one language other than English is used at home, please select the one that is used most often (dropdown)

How many adults aged 18+ normally reside in your household? (dropdown)

How many children aged from 6 to 17 years normally reside in your household? (dropdown)

How many children aged from birth to five years normally reside in your household? (dropdown)

What is your annual household income before tax? This is the total income of all people who live in your household.

My annual household income before tax is (dropdown)

Page 22

Have you ever/are you currently participating in any programs or services to support healthy behaviours and/or growth in any of your children?

If so, can you please briefly describe this program or service? (free text)

End of survey proforma – redirection to PureProfile

Supplementary File 3 - Attributes and levels

- **Cost to the taxpayer (additional cost to you per year, paid as an increase in income taxes)**

A non-systematic search of the cost of early childhood obesity prevention interventions conducted in children aged from birth to five years was conducted, based on key pieces of literature.(Table S3.1) Costs associated with policy level interventions (that might impact this age group, but also broader age groups) or treatment interventions were not included.

Table S3.1 – Examples of costs of early childhood obesity prevention interventions

Intervention	Country	Intervention duration (months)	Reference	Total cost per participant	Total cost per participant (2022 prices*)	Cost per participant per year (2022 prices)^
CHAT SMS	Aus	24	[5]	$80 (AUD2018)	$92	$46
POI-Sleep	Aus	6	[6, 7]	$184 (AUD2018)	$211	$211
Infant	Aus	18	[7]	$331 (AUD2018)	$379	$253
CHAT Telephone	Aus	24	[5]	$394 (AUD2018)	$451	$226
POI-FAB[#]	NZ/Aus	24	[6, 7]	$429 (AUD2018)	$492	$246
Nourish	Aus	16	[7]	$458 (AUD2018)	$524	$393
Infant	Aus	18	[8]	Approx. $500 (AUD2010)	$675	$450
POI combination[#]	NZ/Aus	24	[6]	$601 (AUD2018)	$689	$345
Healthy Beginnings	Aus	24	[7]	$1135 (AUD2018)	$1301	$651
Healthy Beginnings	Aus	24	[7]	$1309 (AUD2018)	$1676	$838
Primrose	Sweden	39	[9]	EUR342 (EUR2015)	$592	$182
Baby Milk	UK	6	[10]	GBP323 (GBP; price year not stated, assumed 2015)	$778	$778

Table notes: * Adjusted using the consumer price index. Foreign currencies were adjusted using currency conversion rates for 1 June in price year. ^ Assuming linearity of cost accrual over time period of intervention. * The Prevention of

Overweight in Infancy trial was conducted in New Zealand, but costed in Australian dollars. AUD= Australian dollars. Aus= Australia. CHAT SMS= Communicating Healthy Beginnings Advice by Telephone- short message service arm. CHAT Telephone= Communicating Healthy Beginnings Advice by Telephone- telephone arm. NZ= New Zealand. POI combination= Prevention of Overweight in Infancy- combination arm. POI FAB= Prevention of Overweight in Infancy- food, activity, breastfeeding arm.

From the estimates in Table S2.1, the mean intervention cost per child per year was estimated at AUD385.

Number of Australian taxpayers (on salary or wages) in 2020-21= 11,900,967 [11]

Number of Australian children aged from birth to five years in 2022= 940,925 [12]

Participation rates in early childhood obesity prevention interventions are unknown, and likely to vary from intervention to intervention. To inform the levels for the cost attribute in the DCE the cost per taxpayer was estimated, assuming participation rates of 10%, 50%, 100% of the eligible population.(Table S3.2)

Table S3.2 – Cost per taxpayer estimations

	Total cost per year	Cost per taxpayer per year
Cost if 10% of the eligible population participate	$36,204,603	$3
Cost if 50% of the eligible population participate	$181,023,013	$15
Cost if 100% of the eligible population participate	$362,046,025	$30

These estimates, and estimates from the literature,[13] were used to inform plausible ranges of cost per taxpayer paid as an increase in income taxes. Levels of cost must extend beyond the cost that is currently paid because the actual cost may not represent an individual's maximum willingness-to-pay.[14] Final levels for inclusion in the DCE were:

$6 per year (approx. $0.25 per fortnight)

$12 per year (approx. $0.50 per fortnight)

$120 per year (approx. $5 per fortnight)

$240 per year (approx. $10 per fortnight)

- **Who can participate in the initiative**

Any child aged from birth to five years

Specific children aged from birth to five years

A plain language definition of the term “specific children” was provided:

Specific children – For example, children living in low socioeconomic areas or children from families that speak languages other than English at home.

Outcome attributes:

Outcomes for selection into the DCE were informed by the Core Outcome Set for the Early Prevention of Obesity in Childhood (COS EPOCH).[15] The development of the COS EPOCH involved a scoping review, a three-round Delphi study and a consensus meeting. Round 3 Delphi study results by stakeholder group, ranked in order of highest degree of importance as perceived by parents/caregivers are in Table S2.3.

Table S3.3 – Round 3 Delphi study results [15]

OUTCOME	HC	COMM	PARENTS	FUND	ACAD	AVG
Child diet quality	8.5	8.8	**8.8**	8.3	8.0	**8.5**
Child PA	8.0	8.0	**8.8**	8.4	8.1	**8.3**
Child wellbeing	7.2	7.8	**8.8**	7.7	6.9	7.7
Child time spent sedentary	8.1	8.5	**8.5**	8.2	7.8	**8.2**
Child dietary intake	8.3	8.3	**8.3**	8.6	7.7	**8.2**
SB/PA home enviro	7.6	8.3	**8.3**	8.2	7.5	8.0
ECEC enviro	7.3	8.0	8.2	8.1	7.2	7.7
Child F&V intake	7.9	9.0	8.2	8.0	7.3	**8.1**
Household food security	8.5	8.3	8.0	8.5	7.9	**8.2**
Family meal enviro	8.0	8.3	8.0	7.7	7.6	7.9
Parent nutrition parenting practices	8.2	7.9	8.0	7.3	7.1	7.7
Infant tummy time	6.7	7.8	7.8	7.3	6.3	7.2
Food environment	7.8	8.0	7.8	8.7	7.8	8.0
Child screen time	7.5	8.0	7.7	8.3	7.8	7.9
Child sleep duration	7.1	7.4	7.7	6.9	7.2	7.3
Parent PA parenting practices	7.3	8.0	7.6	7.3	6.7	7.4
Child unhealthy food intake	8.0	7.7	7.5	8.1	7.1	7.7
Child unhealthy beverages intake	8.5	8.0	7.5	8.2	7.6	8.0
Child meal patterns	7.3	8.0	7.3	7.6	6.9	7.4
Child weight-based anthro	8.3	7.8	7.3	8.2	8.3	8.0
Parent sleep parenting practices	6.7	7.8	7.2	6.8	6.3	7.0
Economic eval	7.3	7.1	6.2	6.9	7.1	6.9

HC=Healthcare professionals. Comm= Community or organisational stakeholders. Fund= Policy makers or funders. Acad= Academics. Top six ranked most important across all stakeholder groups are bolded.

Outcomes were selected based on the following criteria:

- Outcome at the individual child level.

- Outcome does not have conceptual cross-over (e.g. diet quality or intake or fruit and vegetable intake). Where conceptual cross-over existed, a higher order of wording for the outcome was adopted (e.g. diet quality or intake worded as diet).
- From highest degree of importance to lowest.
- Outcome was reworded into plain language for consideration for inclusion in the DCE. Plain language wording of outcomes was undertaken so that participants could easily comprehend the outcome, and aimed to avoid jargon and ambiguous language.

This resulted in selection of attributes for the DCE:

- **Effect on the child's diet**

No effect

Healthier foods and drinks are consumed

- **Effect on how active the child is**

No effect

Healthier amount of time being active

- **Effect on the child's wellbeing**

No effect

Improves wellbeing

- **Effect on the child's growth**

No effect

Healthier growth pattern

Supplementary File 4 - The ESTIMATE checklist [16]

ESTIMATE	Recommendation	Page no.
Estimates	Describe the choice of parameter estimates resulting from the model appropriately and completely, including<br>▪ Whether each variable corresponds to an effects-coded level, a dummy-coded level, or a continuous change in levels<br>▪ Whether each variable corresponds to a main effect or interaction effect<br>▪ Whether continuous variables are linear or have an alternative functional form	8-10
Stochastic	Describe the stochastic properties of the analysis, including<br>▪ The statistical distributions of parameter estimates<br>▪ The distribution of parameter estimates across the sample (preference heterogeneity)<br>▪ The variance of the estimation function, including systematic differences in variance across observations (scale heterogeneity)	8-10
Trade-offs	Describe the trade-offs that can be inferred from the model, including<br>▪ The magnitude and direction of the attribute-level coefficients<br>▪ The relative importance of each attribute over the range of levels included in the experiment<br>▪ The rate at which respondents are willing to trade off among the attributes (marginal rate of substitution)	10-15
Interpretation	Provide interpretation of the results taking into account the properties of the statistical model, including<br>▪ Conclusions that can be drawn directly from the results<br>▪ Applicability of the sample, including subgroups or segments, to the population of interest<br>▪ Limitations of the results	10-15
Method	Describe the reasons for selecting the statistical analysis method used in the analysis, including<br>▪ Why the method is appropriate for analyzing the data generated by the experiment<br>▪ Why the method is appropriate for addressing the underlying research question<br>▪ Why the method was selected over alternative methods	8-10
Assumptions	Describe the assumptions of the model and the implications of the assumptions for interpreting the results, including<br>▪ Assumptions about the error distribution	8-10

	▪ Assumptions about the independence of observations<br>▪ Assumptions about the functional form of the value function	
Transparent	Describe the study in a sufficiently transparent way to warrant replication, including descriptions of<br>▪ The data setup, including handling missing data<br>▪ The estimation function, including the value function and the statistical analysis method<br>▪ The software used for estimation	8-10
Evaluation	Provide an evaluation of the appropriateness of the statistical analysis method to answering the research question, including<br>▪ The goodness of fit of the model<br>▪ Sensitivity analysis of the model specification<br>▪ Consistency of results estimated using different methods	10-15

Supplementary File 5 – MNL model results

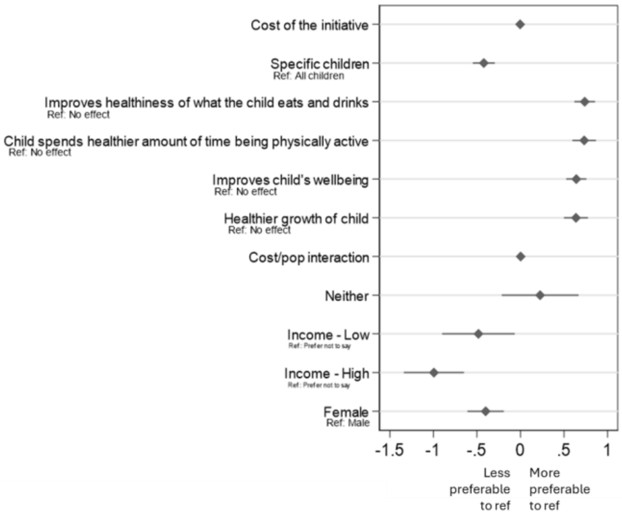

Attribute	Coefficient	SE	P value
**Cost of initiative**			
(paid for by government through taxation or other revenue)	-.00417	.00036	.0000***
**Target population**			
All children (universal)(ref)			
Specific children	-.42112	.06433	.0000***
**Child's diet**			
No effect on what the child eats and drinks (ref)			
Improves the healthiness of what the child eats and drinks	.73747	.06045	.0000***
**How active the child is**			
No effect on how physically active the child is (ref)			
Child spends a healthier amount of time being physically active	.73145	.06900	.0000***
**Wellbeing**			
No effect on the child's wellbeing (ref)			
Improves the child's wellbeing	.64091	.05904	.0000***
**Growth**			
No effect on the child's growth (ref)			
Healthier growth of the child	.63705	.07016	.0000***
**Cost-population**	.00187	.00049	.0001***
**Neither**	0.22520	.22520	.3173
**Income**			
Prefer not to say (ref)			
Low	-.48302	.21275	.0232**
High	-.99447	.17615	.0000***
**Gender**			
Male (ref)	-.40004	.10650	.0002***
Female			

Figure 2 – Forest plot and multinomial logit model analysis results

Figure notes: ref= reference. ***= significant at 1% level; **= significant at 5% level.

Marginal willingness to pay for the MNL analysis was estimated as the ratio of the change in marginal utility of attribute *k* to the change in marginal utility of the cost attribute [17], using the Krinsky and Robb method to estimate 95% confidence intervals.[18]

Table 5.1 – Willingness to pay estimates for each unit increase in outcome attribute, MNL analysis

Outcome attributes	Willingness to pay ($AUD)	95% CI
Initiatives aimed at the general population		
Effect on the child's diet	$177.05***	$141.68 - $212.41
Effect on how active the child is	$175.60***	$138.18 - $213.03
Effect on the child's wellbeing	$153.87***	$118.74 - $188.99
Effect on the child's growth	$152.94***	$112.82 to $193.06
Initiatives aimed at specific children		
Effect on the child's diet	$321.68***	$217.03 - $426.32
Effect on how active the child is	$319.05***	$213.49 - $424.61
Effect on the child's wellbeing	$279.56***	$190.31 - $368.80
Effect on the child's growth	$277.88***	$188.45 - $367.31

Table notes: AUD= Australian dollars. ***= significant at 1% level. 95% CI= 95% confidence interval.

Supplementary File 6 -Dummy coding of demographic variables

Highest level of educational attainment<br>Prefer not to say (ref)<br>1= year 11 or below, high school certificate (year 12), certificate/diploma<br>2= Undergraduate degree, masters degree, PhD
Annual household income before tax<br>Prefer not to say (ref)<br>1= $0 or nil income, $1 to $25,999 ($1-$499 per week), $26,000 to $51,999 ($500-$999 per week)<br>2= $52,000 to $103,999 ($1,000-$1,999 per week),$104,000 to $155,999 ($2,000-$2,999 per week, $156,000 to $207,999 ($3,000-$3,999 per week), $208,000 to $259,999 ($4,000-$4,999 per week, $260,000 to $311,999 ($5,000-$5,999 per week), $312,000 to $415,999 ($6,000-$7,999 per week), $416,000 or more ($8,000 or more per week)
Do you use a language other than English at home?<br>No, only English (ref)<br>1= Yes, all languages

Supplementary File 7 – Secondary analysis incorporating attitudinal variables

Table 6.1 – Attitudinal responses from survey participants (n=466)

	**Count (%)**
**Childhood obesity is serious**	
Do not agree at all	2 (0.5%)
Not sure	14 (3%)
Somewhat agree	82 (17%)
Agree	191 (41%)
Strongly agree	177 (38%)
**Childhood obesity can cause harm in childhood**	
Do not agree at all	2 (0.5%)
Not sure	10 (2%)
Somewhat agree	57 (12%)
Agree	181 (39%)
Strongly agree	216 (46%)
**Childhood obesity can lead to life-threatening diseases in later life**	
Do not agree at all	4 (1%)
Not sure	15 (3%)
Somewhat agree	53 (11%)
Agree	165 (35%)
Strongly agree	229 (49%)
**How worried are you about your child (aged from birth to five years) experiencing overweight or obesity**	
Not worried at all	176 (38%)
A little bit worried	105 (23%)
Moderately worried	95 (20%)
Very worried	55 (12%)
Extremely worried	35 (8%)
**How worried in general should parents be about children experiencing overweight or obesity**	
Not worried at all	8 (2%)
A little bit worried	74 (16%)
Moderately worried	163 (35%)
Very worried	136 (29%)
Extremely worried	85 (18%)
**How supportive are you of the government imposing taxes to fund new initiatives such as early childhood healthy lifestyle initiatives**	
Strongly opposed	24 (5%)
Opposed	49 (10%)
Somewhat supportive	167 (36%)
Supportive	147 (32%)
Strongly supportive	79 (17%)

* May not sum to 100 due to rounding

To explore how attitudes are correlated with choice in a secondary analysis, attitudinal variables were added to the model if they improved model fit and were statistically significant ($p<0.05$).

The utility function for this secondary analysis was specified as:

U(Initiative 1)= β_1Cost + β_2Popn + β_3Diet + β_4Activity + β_5Wellbeing + β_6Growth + β_7Cost-population

U(Initiative2)= β_1Cost + β_2Popn + β_3Diet + β_4Activity + β_5Wellbeing + β_6Growth + β_7Cost-population

U(Neither)= β_8 + β_9Income(low) + β_{10}Income(high) + β_{11}Female + β_{12}Worried + β_{13}Supportive

Table S6.1 – Mixed multinomial logit model analysis results, including statistically significant attitudinal variables

Attribute	Coefficient	Standard error	P value
**Random parameters in utility functions**			
**Cost of initiative**<br>(paid for by government through taxation or other revenue)	-.00789	.00062	.0000***
**Target population**<br>All children (universal)(ref)<br>Specific children	-.72198	.10451	.0000***
**Child's diet**<br>No effect on what the child eats and drinks (ref)<br>Improves the healthiness of what the child eats and drinks	1.10929	.09647	.0000***
**How active the child is**<br>No effect on how physically active the child is (ref)<br>Child spends a healthier amount of time being physically active	1.10913	.10503	.0000***
**Wellbeing**<br>No effect on the child's wellbeing (ref)<br>Improves the child's wellbeing	.97323	.08900	.0000***
**Growth**<br>No effect on the child's growth (ref)<br>Healthier growth of the child	.93982	.11274	.0000***
**Cost-population**	.00204	.00048	.0003***
**Non-random parameters in utility functions**			
**Neither**	3.51912	.28228	.0001***
**Income**<br>Prefer not to say (ref)<br>Low<br>High	 <br>-1.156551<br>-1.93229	 <br>.85552<br>.69883	 <br>.1764<br>.0057**
**Gender**			

Male (ref)<br>Female	-.40041	.32705	.2208
**How worried parents should be about their child experiencing overweight or obesity**<br>1= not worried at all<br>2= a little bit worried<br>3= moderately worried<br>4= very worried<br>5= extremely worried	-.73410	.22351	.0010**
**Support for the government imposing taxes to fund new initiatives such as early childhood healthy lifestyle initiatives**<br>1= strongly opposed<br>2= opposed<br>3= somewhat supportive<br>4= supportive<br>5= strongly supportive	-.47731	.16637	.0041***

Table notes: ref=reference

REFERENCES

1. Bridges, J.F., A.B. Hauber, D. Marshall, A. Lloyd, L.A. Prosser, D.A. Regier, et al., *Conjoint analysis applications in health--a checklist: a report of the ISPOR Good Research Practices for Conjoint Analysis Task Force.* Value Health, 2011. **14**(4): p. 403-13.
2. Ride, J., I. Goranitis, Y. Meng, C. LaBond, and E. Lancsar, *A Reporting Checklist for Discrete Choice Experiments in Health: The DIRECT Checklist.* PharmacoEconomics, 2024. **42**(10): p. 1161-1175.
3. Qualtrics XM. *Qualtrics XM*. 2023; Available from: www.qualtrics.com.
4. ChoiceMetrics, *Ngene v1.2.1*. 2018: Sydney.
5. Killedar, A., L.M. Wen, E.J. Tan, S. Marshall, S. Taki, L. Buchanan, et al., *Economic evaluation of the Communicating Healthy Beginnings Advice by Telephone trial for early childhood obesity prevention.* Obesity, 2022. **30**(11): p. 2256-2264.
6. Tan, E.J., R.W. Taylor, B.J. Taylor, V. Brown, and A.J. Hayes, *Cost-Effectiveness of a Novel Sleep Intervention in Infancy to Prevent Overweight in Childhood.* Obesity, 2020. **28**(11): p. 2201-2208.
7. Brown, V., E.J. Tan, A. Hayes, L. Baur, K. Campbell, R. Taylor, et al., *Cost comparison of five Australasian obesity prevention interventions for children aged from birth to two years.* Pediatric Obesity, 2020. **15**(12): p. e12684.
8. Campbell, K.J., S. Lioret, S.A. McNaughton, D.A. Crawford, J. Salmon, K. Ball, et al., *A Parent-Focused Intervention to Reduce Infant Obesity Risk Behaviors: A Randomized Trial.* Pediatrics, 2013. **131**(4): p. 652-660.
9. Döring, N., N. Zethraeus, P. Tynelius, J. de Munter, D. Sonntag, and F. Rasmussen, *Economic Evaluation of PRIMROSE—A Trial-Based Analysis of an Early Childhood Intervention to Prevent Obesity.* Frontiers in Endocrinology, 2018. **9**.
10. Rajalakshmi, L., J.S. Stephen, W. Fiona, S. Annie, H. Wendy, I. Lisa, et al., *Randomised controlled trial of a theory-based behavioural intervention to reduce formula milk intake.* Archives of Disease in Childhood, 2018. **103**(11): p. 1054.
11. Australian Taxation Office. *Taxation statistics 2020-21*. 2023 [cited 2023 12 September]; Available from: https://www.ato.gov.au/about-ato/research-and-statistics/in-detail/taxation-statistics/taxation-statistics-2020-21/.
12. Australian Bureau of Statistics. *National, state and territory population*. 2022 [cited 2023 12 September]; Available from: https://www.abs.gov.au/statistics/people/population/national-state-and-territory-population/dec-2022#data-downloads.
13. Lancsar, E., J. Ride, N. Black, L. Burgess, and A. Peeters, *Social acceptability of standard and behavioral economic inspired policies designed to reduce and prevent obesity.* Health Economics, 2022. **31**(1): p. 197-214.
14. Ryan, M., *Using conjoint analysis to take account of patient preferences and go beyond health outcomes: an application to in vitro fertilisation.* Soc Sci Med, 1999. **48**(4): p. 535-46.
15. Brown, V., M. Moodie, M. Sultana, K.E. Hunter, R. Byrne, A.L. Seidler, et al., *Core outcome set for early intervention trials to prevent obesity in childhood (COS-EPOCH): Agreement on "what" to measure.* International Journal of Obesity, 2022. **46**(10): p. 1867-1874.
16. Hauber, A.B., J.M. González, C.G.M. Groothuis-Oudshoorn, T. Prior, D.A. Marshall, C. Cunningham, et al., *Statistical Methods for the Analysis of Discrete Choice Experiments: A Report of the ISPOR Conjoint Analysis Good Research Practices Task Force.* Value in Health, 2016. **19**(4): p. 300-315.

17. Lancsar, E. and J. Louviere, *Conducting discrete choice experiments to inform healthcare decision making: a user's guide.* Pharmacoeconomics, 2008. **26**(8): p. 661-77.
18. Hensher, D.A., J.M. Rose, and W.H. Greene, *Applied Choice Analysis*. Vol. 2nd edition. 2015, United Kingdom: Cambridge University Press.
